# How I do it: exoscopic disconnection of anterior fossa dural arteriovenous fistulae

**DOI:** 10.1007/s00701-025-06493-9

**Published:** 2025-03-18

**Authors:** Sergio García-García, Hrvoje Barić, Anni Pohjola, Martin Lehecka

**Affiliations:** 1 Department of Neurosurgery, Helsinki University Hospital, Helsinki, Finland; 2 Department of Neurosurgery, University Hospital Center Zagreb, Zagreb, Croatia

**Keywords:** Dural arteriovenous fistula, Exoscope, Surgery, Minimally invasive

## Abstract

**Background:**

Brain Dural Arteriovenous Fistulae (DAVF) are acquired abnormal connections between dural arteries and cerebral veins or venous sinuses. Disconnection of the pathological shunt is recommended for high-grade fistulae and cases with intolerable symptoms or previous bleedings. Surgical disconnection remains the preferred method for anterior fossa DAVF.

**Method:**

Microsurgical disconnection of anterior fossa DAVF is performed with the assistance of a robotic exoscope. Intraoperative aniography is implemented to confirm the exclusion of DAVF.

**Conclusion:**

The exoscope provides excellent lighting and magnification in challenging surgical fields improving surgeon's ergonomics and enabling tailored, minimally invasive approaches without compromising procedural safety or effectiveness.

**Supplementary Information:**

The online version contains supplementary material available at 10.1007/s00701-025-06493-9.

## Introduction

Brain Dural Arteriovenous Fistulae (DAVF) are acquired abnormal connections between dural arteries and cerebral veins or venous sinuses. These fistulae can present with a diverse range of symptoms, from incidental findings to tinnitus, hydrocephalus, cognitive impairment, movement disorders, or intracranial bleeding [6]. DAVF that drain directly into cortical veins pose a higher risk of bleeding, particularly when these veins are dilated [3].

The primary goal of treatment is to disconnect the pathological shunt, achievable through either endovascular or open surgery. While endovascular therapies are the cornerstone for managing most DAVF, surgical disconnection remains the optimal approach for tentorial DAVF and is the preferred method for anterior cranial fossa fistulae [7].

Recently, the exoscope has gained recognition as a valuable alternative to the traditional surgical microscope [2, 4]. It offers significant advantages, including higher magnification, superior illumination in deep and narrow surgical corridors, and improved ergonomics, even in fields with extreme working angles [1, 2, 4].

This case highlights the utility of the exoscope, demonstrating its capacity to provide exceptional lighting and magnification in challenging surgical fields characterized by depth and constrained angles. These features improve the surgeon's ergonomics and enable a tailored, minimally invasive approach without compromising procedural safety or effectiveness.

Written informed consent for the procedure and the use of anonymized surgical and radiological files for publication was obtained from the patient in accordance with the guidelines of the local Institutional Review Board.

## Relevant surgical anatomy

**Fig. 1:**
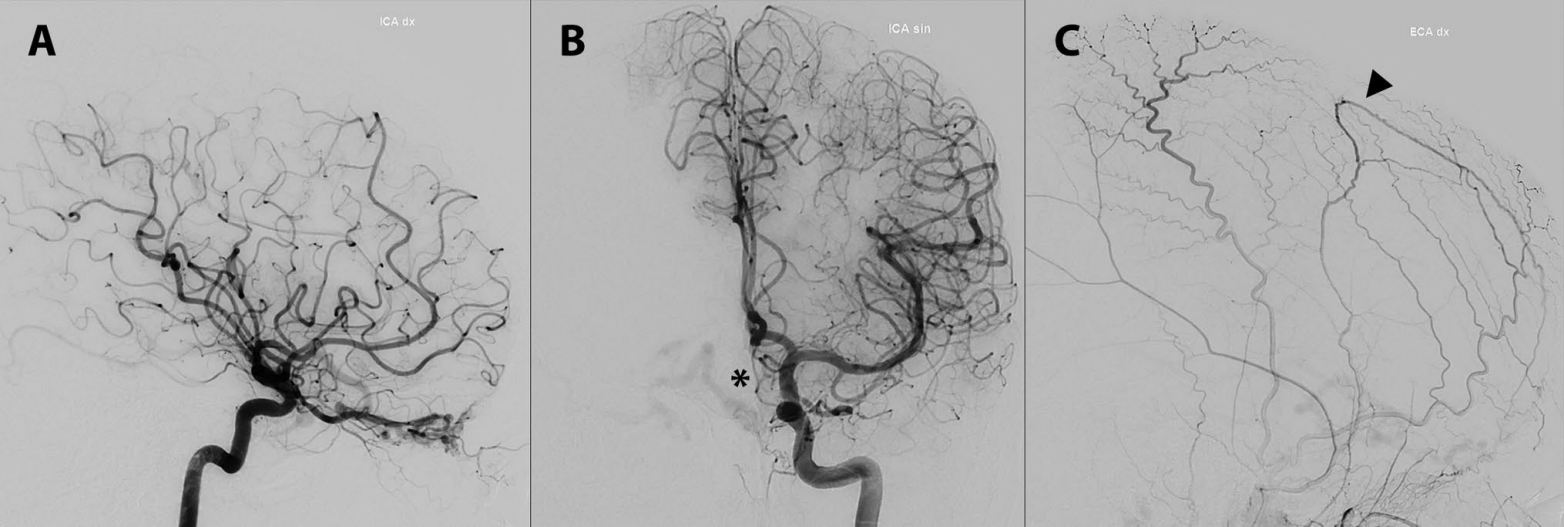
Left anterior Dural Arteriovenous fistula fed bilaterally by right (**A**) and left (**B**) ethmoidal arteries and by a frontal branch of the middle meningeal artery (**C**)

**Fig. 2:**
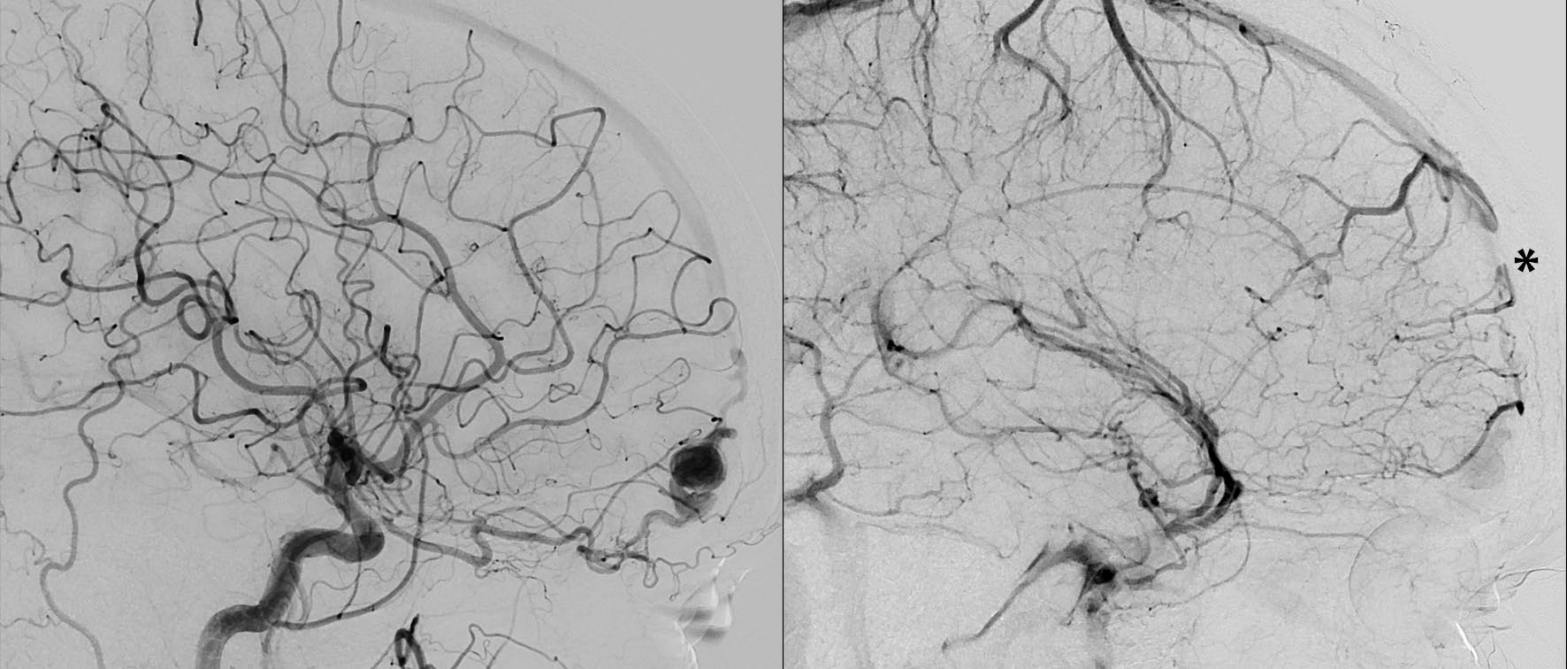
Anterior Dural Arteriovenous fistula Type IV of Cognard classification displaying and enlarged draining vein and an atresic anterior third of the superior sagittal sinus (*)

Anterior fossa DAVF are typically located near the midline, in a paramedian plane at the base of the frontal lobe, with the fistulous point often continuing into cortical veins within the orbitofrontal gyri or the gyrus rectus. They are commonly supplied by the ethmoidal arteries, which originate from the ophthalmic artery. Alternatively, they may be supplied by the angular branch of the facial artery, the middle meningeal artery, or branches of the internal maxillary artery [5] (Fig. 1). Venous drainage frequently occurs into cortical veins such as the frontal, orbitofrontal, or frontopolar veins, most typically draining into the superior sagittal sinus, which may exhibit some degree of atresia in its anterior portion [6] (Fig. 2). Additionally, retrograde venous drainage into the sphenoparietal sinus and the cavernous sinus may also occur [5].

## Description of the technique

The patient is positioned supine with the head extended and rotated 60° to the contralateral side of the DAVF (Video 1). This positioning facilitates passive retraction of the frontal lobe and provides a direct view into the midline. A curvilinear skin incision is marked starting 1 cm anterior to the tragus and 1 cm above the zygoma, extending behind the hairline to approximately the midline. The skin is infiltrated with a combination of lidocaine, ropivacaine, and adrenaline. The skin is incised, and the galea and periosteum are bluntly dissected. The muscle is incised and dissected anteriorly, leaving a cuff at the temporal line for suturing.

A 2.5 cm lateral supraorbital approach is performed, with approximately two-thirds of the bone flap located beneath the temporal muscle. The dura is opened with a caudal base, and dural tack-up sutures are placed to maintain a clean surgical field and optimize exposure.

**Fig. 3:**
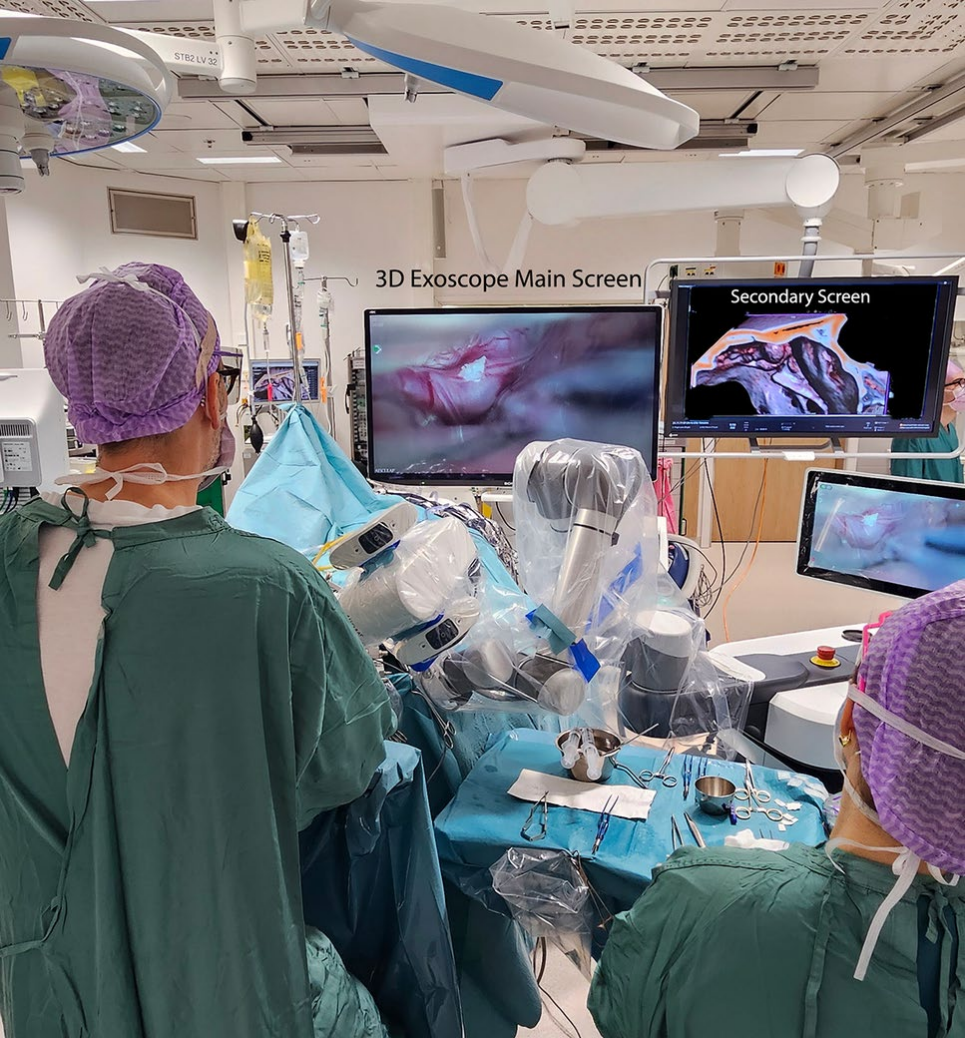
Surgical setting. The exoscope camera is adjusted remotely while both hands are kept in the field to maintain the exposure and control over the structures. The surgeon works in a straight and steady position while the exoscope is angled or tilted to provide an optimal vision of the midline. Despite the extreme angulation the surgeon’s posture remains neutral. Ergonomy is one of the main advantages of exoscopic surgery

A subfrontal arachnoid dissection plane is developed using dynamic retraction with two surgical instruments, allowing gradual advancement toward the fistulous point while the exoscope is remotely manipulated using the foot pedal (Fig. 3). The olfactory nerve serves as a reliable landmark to guide dissection toward the fistulous point near the cribriform plate.

Microsurgical techniques are employed to precisely dissect the fistulous point, which can be confirmed intraoperatively using indocyanine green (ICG) angiography. Dural tributaries are coagulated, and the fistula is disconnected either by clipping or by coagulation and excision as close to its origin as possible.

Dura is closed with running suture. The bone flap is secured with two craniofixes, and bone dust is used to fill defects and promote ossification. The muscle and subcutaneous layers are closed with resorbable continuous sutures, and the skin is closed with staples. An intraoperative digital subtraction angiography (DSA) is performed to confirm complete exclusion of the DAVF.

## Indications

DAVF treatment is recommended for cases with intolerable symptoms, previous bleedings, or angiographic features indicative of high-grade fistulae(Borden III or Cognard > 2b) [8]. The herein described technique can be applied to any lesion in the anterior fossa. Nonetheless,the presented case (Video) highlights the advantages of this approach in a complex setting, demonstrating how exoscope-guided surgery enables a small tailored craniotomy, optimizing microsurgical principles and cosmetic results while maintaining safety and procedural effectiveness.

## Limitations

Small surgical approaches require optimal conditions, including adequate brain relaxation and clear identification of anatomical landmarks. The described technique may not be ideal in cases involving ruptured anterior fossa DAVF with significant intraparenchymal hemorrhage or elevated intracranial pressure. Additionally, anatomical variations such as a high-riding orbital roof or a deeply excavated cribriform plate may obstruct direct visualization of the fistulous point. In such cases, larger craniotomies or additional bony work such as orbital roof drilling may be necessary to achieve adequate exposure.

## How to avoid complications

The application of microsurgical principles is critically important to minimize complications in neurovascular surgery. Maintaining a clean surgical field, providing adequate illumination, and ensuring maximal magnification during arachnoid dissection are essential. The fistulous point must be thoroughly exposed and carefully dissected to achieve a clear view of the anomalous shunt. Confirmation of the fistulous point can be achieved using microdoppler or ICG angiography. To prevent recurrence, exclusion of the DAVF should be performed as close to the dura as possible, with all contributory vessels coagulated or disconnected. It is recommended to use any intraoperative tool(Microdoppler, DSA or ICG) to ensure the complete exclusion of the fistula.

## Specific information for the patient

The primary goal of treatment is to disconnect the pathological shunt, achievable through either endovascular or open surgery. While endovascular therapies are the cornerstone for managing most DAVF, surgical disconnection remains the optimal approach for tentorial DAVF and is the preferred method for anterior cranial fossa fistulae [7].

The secondary goal of every surgery is to reduce its impact on patient’s quality of life. Minimal approaches, thorough bony reconstruction and meticulous closing techniques promote a fast recovery and excellent cosmetic results. Transitory numbness of the forehead might occur as a potential side effect.

## key points summary

- Indication: Surgical disconnection is the preferred treatment for anterior cranial fossa DAVF with direct cortical venous drainage.
- Preoperative Assessment: A thorough DSA evaluation is essential to identify the exact location of the fistulous point and venous drainage pattern, ensuring proper orientation throughout the procedure and minimizing complications.
- Optimal Patient Positioning: Proper head extension and 60° contralateral rotation enhance surgical exposure and optimize working angles.
- Craniotomy: Designing a tailored lateral supraorbital craniotomy with two thirds under the temporal muscle and preserving a muscle cuff facilitates reconstruction and improves cosmetic outcomes.
- The 3D robotic exoscope is most effective when controlled via foot pedal, allowing both hands to remain in the surgical field while utilizing high magnification.
- The olfactory nerve serves as a reliable guide to orientate the dissection toward the cribriform plate, where the fistulous point is often located.
- Fistula Disconnection: All tributaries to the DAVF should be coagulated, and the fistula must be disconnected as close to its origin as possible to prevent recurrence.
- Confirmation of successful DAVF disconnection using ICG, microdoppler, or intraoperative DSA is strongly recommended.
- Approach Limitations: In cases of significant intracranial hemorrhage, high-riding orbital roofs, or deep cribriform plates, additional bony work may be required to achieve adequate exposure.
- Advantages of the Exoscope: The exoscope provides excellent lighting, magnification, and ergonomics, making it particularly beneficial in deep and narrow surgical corridors.

## Supplementary Information

Below is the link to the electronic supplementary material.

ESM 1 Surgical case. The case highlights the surgical benefits of using a robotic exoscope in a complex setting, demonstrating how exoscope-guided surgery enables a tailored, optimized approach to anterior fossa DAVF while maintaining safety and procedural effectivenes. (VIDEO 424 MB)

## Data Availability

No datasets were generated or analysed during the current study.
